# Similar Neural Pathways Control Foraging in Mosquitoes and Worms

**DOI:** 10.1128/mBio.00656-19

**Published:** 2019-04-30

**Authors:** Jogender Singh, Alejandro Aballay

**Affiliations:** a Department of Molecular Microbiology & Immunology, Oregon Health & Science University, Portland, Oregon, USA; Mass General Hospital

**Keywords:** A. aegypti, C. elegans, NPR-1, aversion behavior, bacterial colonization, feeding, neuropeptide Y

## Abstract

Female Aedes aegypti mosquitoes bite human hosts to obtain a blood meal and, in the process, act as vectors for many disease-causing viruses, including the dengue, chikungunya, yellow fever, and Zika viruses. After a complete meal, the female mosquitoes lose attraction to their hosts for several days. New research shows that pharmacological activation of neuropeptide Y-like receptor (NPYLR) signaling elicits host aversion in female mosquitoes. This behavior of mosquitoes shows remarkable similarities to a bacterial-aversion behavior of the nematode Caenorhabditis elegans. Feeding on pathogenic bacteria causes bloating of the gut in C. elegans that leads to activation of NPYLR signaling and bacterial aversion. Several studies suggest that this newly discovered mechanism underlying foraging may be conserved across a large number of species. A better understanding of the regulation of NPYLR signaling pathways could provide molecular targets for the control of eating behaviors in different animals, including human-disease vectors.

## Abstract (precis)

Female Aedes aegypti mosquitoes bite human hosts to obtain a blood meal and, in the process, act as vectors for many disease-causing viruses, including the dengue, chikungunya, yellow fever, and Zika viruses. After a complete meal, the female mosquitoes lose attraction to their hosts for several days.

## PERSPECTIVE

The Aedes aegypti mosquito is the primary vector for many disease-causing viruses, including the dengue, chikungunya, yellow fever, and Zika viruses. The female mosquitoes require blood from a host for the development of eggs and to complete their full reproductive life cycle (1). In the absence of a blood meal, the mosquitoes fail to develop eggs and do not reproduce. Therefore, the female mosquitoes possess a strong host-seeking behavior for a blood meal that depends primarily on sensing carbon dioxide (CO_2_) (1, 2). A single female goes through multiple blood-feeding and egg-laying cycles in her lifetime. Due to the cyclic nature of their feeding behavior, the female mosquitoes serve as effective carriers of disease-causing viruses from infected humans to healthy individuals. Thus, the suppression of the host-seeking behavior or elicitation of host aversion has the potential of being a practical method for reducing the spread of disease-causing viruses. In a recent study, Duvall et al. (3) reported that pharmacological activation of G protein-coupled neuropeptide Y (NPY)-related signaling elicits a host aversion behavior.

Extensive studies have been carried out to understand the blood-feeding behavior of mosquitoes, and they showed that host aversion consists of at least two phases: an early phase involving gut distension from a blood meal (4) and a sustained phase that lasts until the female lays her eggs (5, 6). Previous studies showed that a blood meal to repletion led to enhanced levels of neuropeptides in the hemolymph (7). Injection of hemolymph from blood-fed females or high doses of synthetic peptides that activate NPY-like receptors were sufficient to elicit host aversion in non-blood-fed females (6, 7). These findings suggested that a humoral response involving neuropeptides and activation of an NPY-related pathway plays a role in sustained host aversion in fully fed A. aegypti mosquitoes.

NPY-related signaling pathways are evolutionarily conserved and have been implicated in several biological processes, including foraging, neuronal excitability, stress response, and motivated feeding behavior (8). Small-molecule drugs that possess high affinity to NPY human receptors might bind to mosquito NPY-like receptors and serve as powerful tools to investigate the functional role of NPY-related signaling in host-seeking behavior. Duvall and coworkers (3) performed a targeted drug screen and found that agonists for NPY-related signaling elicit a host aversion behavior even in non-blood-fed mosquitoes. Conversely, an antagonist for the NPY-related signaling elicited host-seeking behavior even in blood-fed mosquitoes. From a screen of all 49 predicted A. aegypti peptide receptors, the authors identified NPY-like receptor 7 (NPYLR7) as the sole target of these drugs. In addition, the investigators identified 9 peptide ligands for NPYLR7, including several Phe-Met-Arg-Phe amides (FMRFamides). These results showed that modulating NPYLR7 activity might affect host-seeking behavior. To identify A. aegypti-specific agonists with no cross-reactivity to human NPY receptors, the authors challenged NPYLR7 with 265,211 unique small molecules. The investigators isolated six highly selective NPYLR7 agonists that inhibit attraction to humans in wild-type mosquitos but not in *NPYLR7* null mutants. Finally, the investigators showed that these drugs were capable of inhibiting biting and blood feeding on a live host and, therefore, suggested a novel approach to control infectious disease transmission by controlling mosquito behavior.

**FIG 1:**
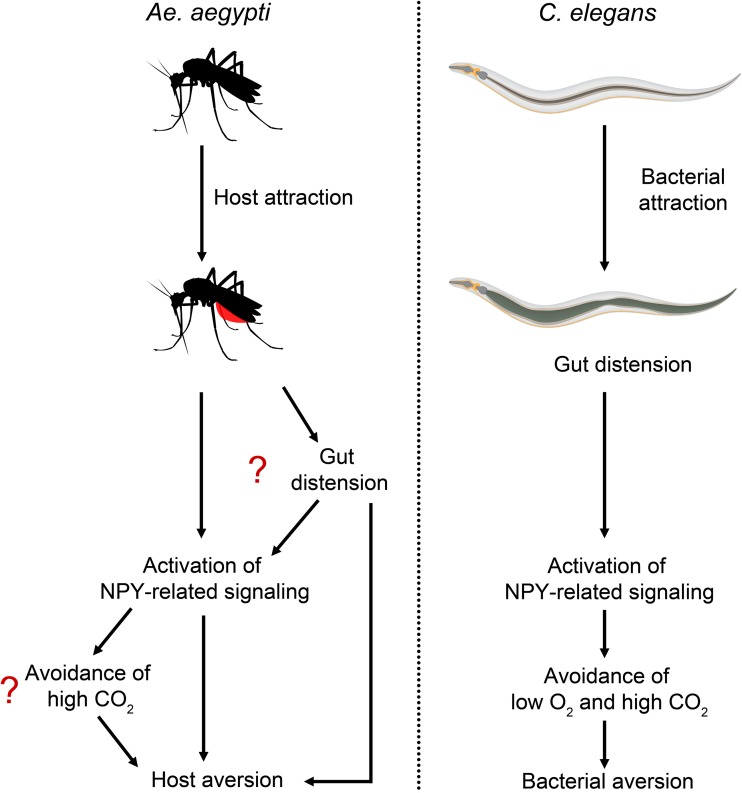
A blood meal to repletion and gut distension caused by bacterial colonization lead to activation of NPY-related signaling in female A. aegypti mosquitoes and C. elegans, respectively. The increased NPY-related signaling is responsible for host aversion behavior in mosquitoes and bacterial-aversion behavior in C. elegans.

This newly described mechanism for induction of host aversion in mosquitoes shows striking similarities to a bacterial-aversion behavior in the model nematode Caenorhabditiselegans (Fig. 1). C. elegans lives in anthropomorphic environments rich in rotting vegetation and decaying fruit, where it is in contact with soilborne microbes, including bacteria that can be used as a food source. A recent study shows that gut distension caused by bacterial ingestion activates a C. elegans NPY-like receptor signal, involving NPR-1 and its FMRF-like peptide ligands, leading to bacterial aversion (9). Inhibition of the NPR-1 signaling pathway elicits avoidance of low CO_2_ and high O_2_, while higher activity of the pathway induces avoidance of high CO_2_ and low O_2_ (10–12). Because bacterial lawns have high CO_2_ and low O_2_ due to bacterial metabolism, NPR-1 signaling integrates this information and helps in the elicitation of bacterial avoidance behavior in C. elegans (10, 13, 14). While mosquitoes seek hosts by their CO_2_ levels and NPY-related signaling induces host aversion, it remains to be studied whether increased NPY-related signaling in blood-fed mosquitoes induces CO_2_ avoidance by a mechanism similar to that of C. elegans (Fig. 1).

An important question that remains unanswered is how NPY-related signaling is activated in mosquitoes after a complete blood meal. In C. elegans, the activation of NPR-1 signaling was independent of colonization by live bacteria and depended on gut distension (9). Given the high similarities between the host aversion behavior of mosquitoes and bacterial-aversion behavior of C. elegans, it is possible that the NPY-related signaling is activated by similar mechanisms in mosquitoes. Indeed, gut distension is shown to be the reason for the early-phase induction of host aversion in mosquitoes. It will be interesting to study whether gut distension leads to the activation of the NPY-related signaling in mosquitoes.

Apart from mosquitoes and C. elegans, a variety of disparate species sense levels of CO_2_ and/or O_2_ in the environment and modulate their behavior accordingly (15). Other blood-feeding insects, such as black flies and tsetse flies, are attracted to CO_2_ and use this signal to seek their human hosts. Similarly, the infective juveniles of the parasitic nematodes *Heterorhabditis bacteriophora* and *Steinernema carpocapsae* are attracted to CO_2_ and use this cue for host seeking (16). CO_2_ may also function as an alarm signal, as CO_2_ emitted by stressed *Drosophila* acts as a signal for other *Drosophila* flies to flee (17). The hawkmoth *Manduca sexta* prefers flowers that emit a high level of CO_2_, suggesting that CO_2_ acts as a proximal signal for nectar (18). Given that CO_2_ and/or O_2_ levels in the environment modulate the behavior of different organisms, it will be important to study whether the molecular mechanisms of sensing CO_2_ and O_2_ are conserved across species. The sensing mechanism of CO_2_ and O_2_ are relatively well studied in the model organisms C. elegans and *Drosophila* (15). This knowledge might act as a primer to expedite the understanding of the underlying molecular mechanisms in disease vectors and parasites.
